# Supplementary material for: Daxx mediated histone H3.3 deposition on HSV-1 DNA restricts genome decompaction and the progression of immediate-early transcription
Source: bioRxiv. 2024 Aug 15:2024.08.15.608064. Preprint. [Version 1] doi: 10.1101/2024.08.15.608064 (PMC11343217; doi:10.1101/2024.08.15.608064)
Supplement: Supplement 1 [file NIHPP2024.08.15.608064v1-supplement-1.pdf]

bioRxiv preprint doi: https://doi.org/10.1101/2024.08.15.608064; this version posted August 15, 2024. The copyright holder for this preprint (which was not certified by peer review) is the author/funder, who has granted bioRxiv a license to display the preprint in perpetuity. It is made available under aCC-BY 4.0 International license.

Supporting information

Fig S1. Histone localization in mock-treated HFt cells. Confocal microscopy images of data presented in Fig 1B. Mock-treated HFt cells were stained for Daxx, HIRA, histones H2A, H2B, H3, or H4 (Channel 1 [Ch.1]; green, as indicated) and PML (red) by indirect immunofluorescence. Nuclei were stained with DAPI (blue). Cut mask (yellow) highlights

bioRxiv preprint doi: https://doi.org/10.1101/2024.08.15.608064; this version posted August 15, 2024. The copyright holder for this preprint (which was not certified by peer review) is the author/funder, who has granted bioRxiv a license to display the preprint in perpetuity. It is made available under aCC-BY 4.0 International license.

regions of colocalization between cellular proteins of interest and PML; weighted colocalization coefficient shown.

Fig S2. Ectopic expression of fluorescently tagged histones in mock-treated HFt cells. HFt cells were stably transduced with lentiviral vectors encoding C-terminally tagged fluorescent (mEmerald; mEm) histones or eYFPnls (negative control) as indicated. (A) Cells were induced to express proteins of interest for 24 h with doxycycline (DOX) prior to whole cell lysate (WCL) collection and western blotting. Membranes were probed for GFP and endogenous (endog.) histones H2A or H3. (B) RPE cells were transfected with plasmids expressing eGFP, H3.1-mEm, or H3.3-mEm for 24 h prior to WCL collection and western blotting. Membranes were probed for GFP and histone H3. (A/B) Molecular mass markers shown. (C to E) HFt cells were DOX induced for 6 h prior to fixation and indirect immunofluorescence staining for PML (red). Nuclei were stained with DAPI (blue). (C) Confocal microscopy images of histone-mEm or eYFPnls localization at PML-NBs. Cut mask (yellow) highlights regions of colocalization between cellular proteins of interest and PML; weighted (w.) colocalization coefficient (coeff.) shown. (D) Quantitation of the percentage of cells that demonstrate histone-mEm or eYFPnls colocalization at PML-NBs. Means and SD shown. (E) Violin plots showing histone-mEm w. colocalization coeff. frequency at PML-NBs: median w. colocalization coeff., solid black line; 25th to 75th percentile range, dotted black lines; coincidence threshold (0.2), dotted grey line; high confidence threshold (0.7), solid grey line. Mann-Whitney *U*-test, *P*-value shown. (D/E) N≥ 150 nuclei per sample condition. (A to E) Data derived from a minimum of three independent experiments. Raw values presented in S1 data.

Fig S3. Localization of fluorescently tagged histones to mitotic cellular chromatin. HFt cells stably transduced with lentiviral vectors encoding C-terminally tagged fluorescent (mEmerald; mEm) histones (as indicated) or eYFPnls (negative control) were induced with doxycycline for 6 h prior to fixation. Nuclei were stained with DAPI (blue). Representative x63 objective lens wide-field confocal microscopy images showing histone-mEm localization in mock-treated HFt cells. Dashed boxes show magnified regions of interest highlighting histone-mEm or eYFPnls localization at mitotic chromatin.

Fig S4. Localization of endogenous histone to nuclear infecting HSV-1 genomes. Confocal microscopy images of data presented in Fig. 1D to G. HFt cells were infected with WT HSV-1^{EdC} (MOI of 1 PFU/cell). Cells were fixed at 90 mpi and stained for Daxx, HIRA, histones H2A, H2B, H3, or H4 (Channel 1 [Ch.1]; green, as indicated) and PML (red) by indirect immunofluorescence. vDNA (red) was detected by click chemistry. Nuclei were stained with DAPI (blue). Cut mask (yellow) highlights regions of colocalization between cellular proteins of interest and vDNA or PML (as indicated); weighted colocalization coefficient shown. Dashed boxes show magnified regions of interest. White arrows highlight regions of colocalization at vDNA.

bioRxiv preprint doi: https://doi.org/10.1101/2024.08.15.608064; this version posted August 15, 2024. The copyright holder for this preprint (which was not certified by peer review) is the author/funder, who has granted bioRxiv a license to display the preprint in perpetuity. It is made available under aCC-BY 4.0 International license.

Fig S5. Localization of endogenous histone H2A/H2B heterodimers to nuclear infecting HSV-1 genomes. (A/B) Confocal microscopy images of data presented in Fig. 1E and F. HFt cells were mock-treated or infected with WT HSV-1^{EdC} (MOI of 1 PFU/cell). Cells were fixed at 90 mpi and stained for heterodimeric histone H2A/H2B (green) using a fluorescently conjugated nanobody and PML (cyan) by indirect immunofluorescence. vDNA (red) was detected by click chemistry. Nuclei were stained with DAPI (blue). Cut mask (yellow) highlights regions of colocalization between cellular proteins of interest and vDNA or cellular chromatin; weighted colocalization coefficient shown. Dashed box shows magnified region of interest. White arrows highlight regions of colocalization at vDNA. (B) Localization of histone H2A/H2B heterodimers to mitotic chromatin in mock-treated HFt cells.

Fig S6. Localization of fluorescent histones to nuclear infecting HSV-1 genomes. HFt cells stably transduced with lentiviral vectors encoding C-terminally tagged fluorescent (mEmerald; mEm) histones or eYFPnls (negative control) (Channel 1 [Ch.1]; green, as indicated) were induced with doxycycline for 6 h prior to infection with WT HSV-1^{EdC} (MOI of 1 PFU/cell). Cells were fixed at 90 mpi and stained for PML (cyan) by indirect immunofluorescence and vDNA (red) by click chemistry. Nuclei were stained with DAPI (blue). Cut mask (yellow) highlights regions of colocalization between cellular proteins of interest or vDNA (as indicated); weighted colocalization coefficient shown. Dashed boxes show magnified regions of interest. White arrows highlight regions of colocalization at vDNA.

Fig S7. Localization of Daxx and endogenous histones to nuclear infecting HSV-1 genomes in NTC and PML KO HFt cells. Confocal microscopy images of data presented in Fig. 5C, D. NTC and PML KO HFt cells were infected with WT HSV-1^{EdC} (MOI of 1 PFU/cell). Cells were fixed at 90 mpi and stained for Daxx, histones H2A, H2B, H3, or H4 (green, as indicated) and PML (cyan) by indirect immunofluorescence. vDNA (red) was detected by click chemistry. Nuclei were stained with DAPI (blue). Cut mask (yellow) highlights regions of colocalization between cellular proteins of interest and vDNA (as indicated); weighted colocalization coefficient shown. White arrows highlight regions of colocalization at vDNA. Dashed boxes show magnified regions of interest.

Fig S8. Localization of histone H3, ATRX, and PML in NTC and Daxx KO HFt cells. Confocal microscopy images of data presented in Fig. 6A. Mock-treated NTC and Daxx KO HFt cells were fixed and stained for PML, ATRX, and histone H3 (green, as indicated) and Daxx (cyan) by indirect immunofluorescence. Nuclei were stained with DAPI (blue). Cut mask (yellow) highlights regions of colocalization between cellular proteins of interest and Daxx (as indicated); weighted (w.) colocalization coefficient (coeff.) shown.

bioRxiv preprint doi: https://doi.org/10.1101/2024.08.15.608064; this version posted August 15, 2024. The copyright holder for this preprint (which was not certified by peer review) is the author/funder, who has granted bioRxiv a license to display the preprint in perpetuity. It is made available under aCC-BY 4.0 International license.

Fig S9. Localization of histones H3 and H4 to nuclear infecting HSV-1 genomes in NTC and Daxx KO HFt cells. Confocal microscopy images of data presented in Fig. 6E, F. NTC and Daxx KO HFt cells were infected with WT HSV-1^{EdC} (MOI of 1 PFU/cell). Cells were fixed at 90 mpi and stained for PML, ATRX, histones H3 or H4 (green, as indicated), and Daxx (cyan) by indirect immunofluorescence. vDNA (red) was detected by click chemistry. Nuclei were stained with DAPI (blue). Cut mask (yellow) highlights regions of colocalization between cellular proteins of interest and vDNA (as indicated); weighted colocalization coefficient shown. White arrows highlight regions of colocalization at vDNA. Dashed boxes show magnified regions of interest.

Fig S10. Daxx restricts the progression of WT HSV-1 IE transcription. (A) HFt cells were mock-treated or infected with WT or ICP0 null-mutant (ΔICP0) HSV-1 (MOI of 3 PFU/cell) in the absence or presence of the proteasome inhibitor MG132 (5 µM). WCLs were collected at the indicated times (h) post-infection (hpi) and analyzed by western blotting. Membranes were probed for ATRX, Daxx, PML, viral IE proteins (ICP0 and ICP4), histone H3, and actin (loading control). Molecular mass markers shown. < denotes the detection of a non-specific viral protein. (B) Independent replicate experiments of data presented in Fig. 8E. NTC and Daxx KO HFt cells were infected with WT HSV-1 (MOI 0.5 PFU/cell). RNA was extracted at the indicated times (minutes post-infection; mpi) and HSV-1 IE transcription (ICP0 and ICP4) quantified by RT-qPCR analysis. Values were normalized to infected NTC cells at 360 mpi. N=3 independent experiments. Means and SD per experiment shown. Raw values presented in S1 data.

S1 Data. Underlying data used for quantitative analysis in this study.
